# Usage of skin care products and risk of rheumatoid arthritis: results from the Swedish EIRA study

**DOI:** 10.1186/ar3749

**Published:** 2012-02-28

**Authors:** Berit M Sverdrup, Henrik Källberg, Lars Klareskog, Lars Alfredsson

**Affiliations:** 1 Rheumatology Unit, Department of Medicine, Karolinska Institutet/Karolinska University Hospital, 171 76 Stockholm, Sweden; 2 Department of Rheumatology, Mälarsjukhuset, 631 88 Eskilstuna, Sweden; 3 Institute of Environmental Medicine, Box 210, Nobelsväg. 13, Karolinska Institutet, 171 79 Stockholm, Sweden

## Abstract

**Introduction:**

The aim of this study was to investigate the association between exposure to cosmetics, often containing mineral oil, and the risk of developing rheumatoid arthritis (RA). The study was performed against the background that occupational exposure to mineral oil has recently been shown to be associated with an increased risk for RA in man, and that injection of or percutaneous exposure to mineral-oil-containing cosmetics can induce arthritis in certain rat strains.

**Methods:**

A population-based case-control study of incident cases of RA was performed among the population aged 18 to 70 years in a defined area of Sweden during May 1996 to December 2003. A case was defined as an individual from the study base, who received for the first time a diagnosis of RA according to the 1987 American College of Rheumatology criteria. Controls were randomly selected from the study base with consideration taken for age, gender and residential area. Cases (*n *= 1,419) and controls (*n *= 1,674) answered an extensive questionnaire regarding environmental and lifestyle factors including habits of cosmetic usage. The relative risk of developing RA was calculated for subjects with different cosmetic usage compared with subjects with low or no usage. Analysis was also performed stratifying the cases for presence/absence of rheumatoid factor and antibodies to citrulline-containing peptides.

**Results:**

The relative risks of developing RA associated with use of cosmetics were all close to one, both for women and men, for different exposure categories, and in relation to different subgroups of RA.

**Conclusion:**

This study does not support the hypothesis that ordinary usage of common cosmetics as body lotions, skin creams, and ointments, often containing mineral oil, increase the risk for RA in the population in general. We cannot exclude, however, that these cosmetics can contribute to arthritis in individuals carrying certain genotypes or simultaneously being exposed to other arthritis-inducing environmental agents.

## Introduction

Rheumatoid arthritis (RA) is a disease that is dependent on genetic as well as environmental factors, as seen from both concordance data in twins and from a number of epidemiological and genetic studies [1,2]. Whereas knowledge of the genetic basis of this disease is rapidly advancing [3-5], there is a scarcity of data on environmental agents that may cause arthritis [6-9]. In particular, very little information exists in humans on environmental factors with a known capacity to induce arthritis in experimental arthritis systems.

Agents that are able to induce experimental arthritis in animals, particularly in rats, include a number of adjuvants originating from microbes such as bacteria, yeast and viruses [10,11] as well as from other sources such as mineral oils [12-14]. Arthritis may develop in certain strains of rats after exposure to adjuvants both intracutaneously and percutaneously [15]. The exact mechanisms involved in the pathogenesis of these adjuvant arthritis models are still not completely understood, but we know that mineral oil can activate cells within the lymph nodes without causing any simultaneous apparent inflammatory reaction in the skin [16]. In the context of mineral oil, we investigated the arthritogenic capacity of common cosmetics that are known to contain high amounts of mineral oils, and we observed that several such cosmetics could induce arthritis in the DA strain of rats when administered subcutaneously as well as percutaneously [16].

It has not been established whether similar mechanisms - that is, polyarthritis induced by simple adjuvants - are operative also in human arthritis, although case reports on arthritis development after adjuvant exposures suggested that rodents and humans might both react to adjuvants with arthritis development [17]. We recently, however, used information obtained from a large ongoing case-control study (Epidemiological Investigation of Rheumatoid Arthritis (EIRA)) to describe an association between occupational exposure to mineral oil and development of RA [18].

The combined observations from rodents on an arthritogenic capacity of cosmetics containing mineral oil, and the observation on mineral oils as a risk factor also for human RA, mandated an investigation of whether usage of cosmetics, often containing mineral oil, is associated with an increased risk for RA. We also used the large EIRA study for this purpose, utilising a series of detailed questions to cases and controls on previous use of various cosmetics as skin creams, ointments and body lotions. The level of exposure to mineral oil via cosmetic use is probably essentially lower than the level we previously analysed in the context of occupational exposure [18], but exposure via cosmetic use is very common, especially among women, and even a small increase in risk of RA might be of importance from a public health perspective.

## Materials and methods

The present study is a population-based case-control study of incident cases of RA among the population aged 18 to 70 years living in the central and southern parts of Sweden during the period May 1996 to December 2003. Ethical permission was obtained from relevant ethical committees and all participants (cases as well as controls) consented to contribute to the study.

### Case identification

A case was defined as a person in the study base who for the first time received a diagnosis of RA according to the 1987 American College of Rheumatology criteria [19]. As described before [20], all potential cases were examined and diagnosed by a rheumatologist at the units entering cases into the study. All rheumatology units linked to the general welfare system in the study area participated in the study as well as almost all of the, very few, privately-run rheumatology units. In total there were 19 reporting clinics, 15 of which were Early Arthritis Clinics [21]. At the start some centres also reported cases that did not satisfy the criteria in order to enable investigations of undifferentiated arthritis, but these subjects were eventually excluded from the study.

Rheumatoid factor (RF) levels were determined locally and reported as RF-positive or RF-negative. Anti-cyclic citrullinated peptide (anti-CCP) antibodies were analysed in a central laboratory (Department of Clinical Immunology, Uppsala University Hospital) with the Immunoscan-RA Mark2 ELISA test (Eurodiagnostica, Malmö, Sweden) [22,23]. A level above 25 U/ml was regarded as positive according to instructions in the kit and validation at the clinical immunology laboratory in Uppsala.

### Selection of controls

For each potential case, a control was randomly selected from the study base matched on age, gender and residential area. The selection of controls was conducted using the national population register, which is continuously updated. If a control declined to participate, was not traceable or reported having RA, a new control was selected using the same principles (see also [20]). Controls selected to cases that were excluded due to not fulfilling the American College of Rheumatology criteria (*n *= 255) remained in the study.

### Data collection

Information about lifestyle and environmental exposures was collected by questionnaire, an identical version given to the cases shortly after they had been informed about the RA diagnosis and sent by mail to the controls. All questionnaires were supposed to be answered at home.

Unanswered or incompletely answered questionnaires were completed by mail or by telephone by purpose-trained persons not connected to the rheumatology clinics. This was done in an identical way for the case and control groups. In all, we identified 1,480 cases and 2,038 controls for selection. Of these, 1,419 cases (1,012 women and 407 men) and 1,674 controls (1,188 women and 486 men) participated in the study, giving a participation rate of 96% for cases and 82% for controls.

### Exposure

The questionnaire contains questions within a wide spectrum regarding personal circumstances including lifestyle factors, occupational exposures, health aspects, socio-economic factors, and demographic data. Specific questions were asked about skin care habits in terms of frequency of usage of cosmetic products as ointments, skin creams and body lotions during the last 5 years. These questions were asked for different parts of the body separately.

A global whole-body exposure measure was constructed by applying different scores according to frequency of use. In this whole-body exposure measure, each part of the body received the same weight (Table 1). For each person, the scores for their five different body regions were added. The whole-body exposure measure was categorised into five different categories (A, 0 to 20; B, 24 to 42; C, 48 to 64; D, 72 to 86; and E, 96 to 120) with the aim to weight the score so that seldom use over the whole body never got the same score as daily use on one single part of the body (Table 2).

**Table 1:** Scoring of cosmetic exposure by frequency of use and body region

Part of body	Daily	Every week	Seldom	Never
Face/neck	24	12	2	0
Hands	24	12	2	0
Legs	24	12	2	0
Feet	24	12	2	0
Body	24	12	2	0

**Table 2:** Exposure frequency of cosmetics, by gender and disease status

		Women						Men					
Cosmetic exposure		Cases		Controls		Total		Cases		Controls		Total	
Class	Score	*n*	%	*n*	%	*n*	%	*n*	%	*n*	%	*n*	%
A	0 to 20	104	10.28	118	9.95	237	10.18	315	77.59	359	74.33	715	75.50
B	24 to 42	178	17.59	174	14.67	376	16.16	53	13.05	78	16.15	140	14.78
C	48 to 64	178	17.59	230	19.39	436	18.74	22	5.42	27	5.59	55	5.81
D	72 to 86	319	31.52	370	31.20	729	31.33	12	2.96	13	2.69	26	2.75
E	96 to 120	233	23.02	294	24.79	549	23.59	4	0.99	6	1.24	11	1.16
Total		1,012		1,186		2,327		406		483		947	

### Potential confounding factors

All results were adjusted for age and residential area according to the principle of control selection. In the analyses, age was categorised into 10 strata (18 to 24, 25 to 29, 30 to 34, 35 to 39, 40 to 44, 45 to 49, 50 to 54, 55 to 59, 60 to 64 and 65 to 70 years of age). Smoking and occupational class were also considered as potential confounding factors. Smoking was categorised into two strata (never smokers and ever smokers) and occupational class was categorised into seven strata (unskilled manual workers, skilled manual workers, assistant nonmanual employees, intermediate nonmanual employees, higher nonmanual employees, self-employed and farmers).

### Statistical analysis

In the analysis, subjects who had been exposed to different categories of use of cosmetics were compared with subjects with no or low use (score 0 to 20) with regard to the incidence of RF-positive RA, RF-negative RA, anti-CCP-positive RA, anti-CCP-negative RA and RA overall, respectively, by calculating the odds ratio together with a 95% confidence interval. We performed matched as well as unmatched analyses of the data. Odds ratios, adjusted for potential confounding factors, were calculated by means of conditional logistic regression in the matched analyses and by means of unconditional logistic regression in the unmatched analyses. We only present results from the unmatched analyses as these were in close agreement with those from the matched analyses but, in general, had higher precision. Odds ratios were interpreted as estimates of relative risks as the study was population based [24]. Results for women and men were analysed separately and in total. Estimates of relative risk were adjusted for potential confounding from age, gender (when appropriate), residential area, smoking and socio-economic class.

All analyses were performed using the Statistical Analysis System (version 8.2; SAS Institute, Stockholm, Sweden).

## Results

Of the 1,419 participating cases in this study, 1,012 were women and 407 were men (mean age at inclusion 50 and 53 years, respectively). Among the female and male cases, respectively, 65.5% and 66.3% were RF-positive and 61% and 62% were anti-CCP-positive. The mean duration of disease at inclusion in the study was 10 months.

Most women were regular users of cosmetics as body lotions and skin creams. Only 10% of female controls were low users. In contrast, most of the male controls (74%) were low users.

When analysing the relationship between exposure to skin care products and risk of developing RA, no increased risk of developing RA in total was observed, neither for women or for men; all observed relative risks were close to 1 (Table 3). When the analysis was stratified with regard to RF status for the cases, still no signs of increased relative risks associated with cosmetic use were observed (Table 4). On the contrary, the most heavily exposed category was associated with a 30% decreased risk of RF-positive RA (relative risk = 0.7, 95% confidence interval = 0.5 to 1.0) (Table 4). A similar pattern was observed with regard to anti-CCP-positive RA, where the most exposed category was also associated with a 30% decreased risk (relative risk = 0.7, 95% confidence interval = 0.5 to 1.0) (Table 5).

**Table 3:** Relative risk of developing rheumatoid arthritis for subjects reporting exposure to cosmetics

Exposure	Number of exposed cases/controls	Relative risk^a^	95% confidence interval	Relative risk^b^	95% confidence interval
Women					
A	104/118	1.0	Reference	1.0	Reference
B	178/174	1.1	0.8 to 1.6	1.0	0.7 to 1.5
C	178/230	0.9	0.6 to 1.2	0.8	0.6 to 1.2
D	319/370	1.0	0.7 to 1.3	0.9	0.6 to 1.2
E	233/294	0.9	0.6-1.2	0.8	0.5 to 1.1
Men					
A	315/359	1.0	Reference	1.0	Reference
B	53/78	0.7	0.5 to 1.1	0.8	0.5 to 1.2
C	22/27	0.8	0.5 to 1.5	0.9	0.5 to 1.6
D	12/13	1.1	0.5 to 2.5	1.1	0.5 to 2.5
E	4/6	0.9	0.2 to 3.2	0.6	0.1 to 2.6
All					
A	419/477	1.0	Reference	1.0	Reference
B	231/252	0.9	0.7 to 1.2	0.9	0.7 to 1.2
C	200/257	0.9	0.7 to 1.2	0.8	0.6 to 1.1
D	331/383	1.0	0.7 to 1.3	0.9	0.7 to 1.2
E	237/300	0.9	0.6 to 1.2	0.8	0.5 to 1.1

Relative risk together with 95% confidence interval of developing rheumatoid arthritis overall for subjects 18 to 70 years old reporting different amounts of exposure to cosmetics compared with those low exposed (A), by gender.

^a^Adjusted for age, living area and gender where appropriate. ^b^Adjusted for age, living area, gender, socio-economic status and smoking where appropriate.

**Table 4:** Relative risk of developing RF-positive and RF-negative rheumatoid arthritis for subjects reporting exposure to cosmetics

	RF^+^RA					RF^-^RA				
Exposure	Number of exposed cases/controls^a^	RR^b^	95% CI	RR^c^	95% CI	Number of exposed cases/controls^a^	RR^b^	95% CI	RR^c^	95% C
	**Women**					**Women**				
A	73/118	1.0	Reference	1.0	Reference	30/118	1.0	Reference	1.0	Reference
B	118/174	1.1	0.7-1.5	1.0	0.6-1.5	60/174	1.3	0.8-2.2	1.2	0.7-2.1
C	115/230	0.8	0.5-1.2	0.7	0.5-1.1	63/230	1.1	0.6-1.7	0.9	0.5-1.7
D	215/370	0.9	0.6-1.3	0.8	0.6-1.2	104/370	1.1	0.7-1.7	1.0	0.6-1.7
E	139/294	0.7	0.5-1.0	0.7	0.4-1.0	94/294	1.2	0.7-2.0	1.0	0.6-1.8
	**Men**					**Men**				
A	212/359	1.0	Reference	1.0	Reference	103/359	1.0	Reference	1.0	Reference
B	31/78	0.6	0.4-1.0	0.7	0.4-1.1	22/78	1.0	0.6-1.6	1.0	0.6-1.8
C	14/27	0.8	0.4-1.6	0.8	0.4-1.6	8/27	0.9	0.4-2.1	1.0	0.4-2.3
D	7/13	1.0	0.4-2.5	1.0	0.4-2.7	5/13	1.3	0.5-4.0	1.3	0.4-4.1
E	3/6	0.8	0.2-3.3	0.5	0.1-2.8	1/6	1.0	0.1-9.0	0.9	0.1-9.1
	**All**					**All**				
A	285/477	1.0	Reference	1.0	Reference	133/477	1.0	Reference	1.0	Reference
B	149/252	0.9	0.6-1.2	0.8	0.6-1.1	82/252	1.1	0.8-1.6	1.1	0.7-1.6
C	129/257	0.8	0.6-1.1	0.7	0.5-1.1	71/257	1.0	0.7-1.6	0.9	0.6-1.5
D	222/383	0.9	0.6-1.2	0.8	0.6-1.2	109/383	1.1	0.7-1.7	1.1	0.7-1.7
E	142/300	0.7	0.5-1.0	0.7	0.5-1.0	95/300	1.2	0.7-1.9	1.0	0.6-1.7

Relative risk (RR) together with 95% confidence interval (CI) of developing rheumatoid factor (RF)-positive rheumatoid arthritis (RA), and RF-negative RA for subjects 18 to 70 years old reporting different amounts of exposure to cosmetics compared with those low exposed (A), by gender. ^a^Information on RF status is missing for one female case in comparison with Table 3. ^b^Adjusted for age, living area and gender where appropriate. ^c^Adjusted for age, living area, gender, socio-economic status and smoking where appropriate.

**Table 5:** Relative risk of developing anti-CCP-positive and anti-CCP-negative rheumatoid arthritis for subjects reporting exposure to cosmetics

	Anti-CCP-positive RA					Anti-CCP-negative RA				
Exposure	Number of exposed cases/controls^a^	RR^b^	95% CI	RR^c^	95% CI	Number of exposed cases/controls^a^	RR^b^	95% CI	RR^c^	95% CI
Women										
A	65/118	1.0	Reference	1.0	Reference	38/118	1.0	Reference	1.0	Reference
B	110/174	1.1	0.7 to 1.7	1.0	0.7 to 1.6	68/174	1.2	0.7 to 1.9	1.1	0.6 to 1.9
C	106/230	0.8	0.6 to 1.2	0.8	0.5 to 1.2	68/230	0.9	0.6 to 1.5	0.9	0.5 to 1.5
D	199/370	0.9	0.6 to 1.3	0.8	0.6 to 1.2	116/370	0.9	0.6 to 1.4	0.9	0.6 to 1.4
E	130/294	0.7	0.5 to 1.1	0.7	0.4 to 1.0	100/294	1.0	0.6 to 1.6	0.9	0.5 to 1.5
Men										
A	197/359	1.0	Reference	1.0	Reference	113/359	1.0	Reference	1.0	Reference
B	27/78	0.6	0.4 to 1.0	0.6	0.3 to 1.0	25/78	1.0	0.6 to 1.6	1.1	0.6 to 1.8
C	12/27	0.7	0.3 to 1.4	0.7	0.3 to 1.4	10/27	1.1	0.5 to 2.5	1.2	0.6 to 2.8
D	7/13	1.1	0.4 to 2.9	1.1	0.4 to 3.0	5/13	1.3	0.4 to 3.7	1.2	0.4 to 3.6
E	4/6	1.3	0.3 to 4.7	0.9	0.2 to 3.8	0/6	n.a.	n.a.	n.a	n.a.
All										
A	162/477	1.0	Reference	1.0	Reference	151/477	1.0	Reference	1.0	Reference
B	137/252	0.9	0.6 to 1.2	0.8	0.6 to 1.1	93/252	1.1	0.7 to 1.5	1.0	0.7 to 1.5
C	118/257	0.8	0.6 to 1.1	0.7	0.5 to 1.1	78/257	1.0	0.6 to 1.4	0.9	0.6 to 1.4
D	206/383	0.9	0.7 to 1.3	0.9	0.6 to 1.2	121/383	0.9	0.6 to 1.4	0.9	0.6 to 1.4
E	134/300	0.8	0.6 to 1.1	0.7	0.5 to 1.0	100/300	0.9	0.6 to 1.4	0.8	0.5 to 1.3

Relative risk (RR) together with 95% confidence interval (CI) of developing anti-cyclic citrullinated peptide-positive rheumatoid arthritis (RA) and anti-CCP-negative RA for subjects 18 to 70 years old reporting different amounts of exposure to cosmetics compared with those low exposed (A), by gender. ^a^Information on anti-CCP status is missing for 12 female and five male cases in comparison with Table 3. ^b^Adjusted for age, living area and gender where appropriate. ^c^Adjusted for age, living area, gender, socio-economic status and smoking where appropriate.

## Discussion

The results of this study provide no evidence that women or men exposed to common cosmetics as skin creams, ointments or body lotions have an increased risk of developing RA.

Our study has the advantage of being a population-based case-control study using only incident cases of newly diagnosed RA, fulfilling the American College of Rheumatology criteria as assessed by a specialist in rheumatology. To minimise recruitment bias, we took advantage of the fact that almost all healthcare in Sweden is provided within the general healthcare system, and that all such units in the area that defined the study base contributed to the study, as did almost all of the few privately-run rheumatology units. Still, some cases may have been unidentified in our study; for instance, cases diagnosed in primary healthcare facilities that were never referred to a rheumatology unit. We know from population-based studies aimed at identifying RA cases directly in primary care, however, that almost all cases of RA in our current Swedish system are indeed referred to rheumatology units. The relatively few unidentified cases would therefore probably not cause a substantial bias in our calculations. The response rate in the study was high, at 96% for cases and 82% for controls, which limits risk for selection bias in this stage.

In our study, information on cosmetic use was based on self-reported data about the frequency of cosmetic use on different parts of the body, as reported in a questionnaire. A possible disadvantage with a case-control study with retrospective collection of exposure data is the risk of misclassification of exposure due to recall bias. Only subjects that received a diagnosis of RA for the first time were included in order to reduce the risk of recall bias; the mean duration between the estimated disease onset and inclusion into the study was 10 months. In light of the observed results, bias due to nondifferential misclassification of cosmetic use could in principle have led to diluted estimates of relative risks and hence could have masked true positive associations. The observation, in our data, of a tendency towards a decreased risk of RA among those with the highest exposure to cosmetics, however, makes unlikely the possibility that nondifferential misclassification of exposure has masked a true positive association between cosmetic use and RA. Still, there is a possibility that differential misclassification may have done so. If cases underreport true exposure to a higher extent than controls, this may indeed be the case. We have no reason to believe that this is the case, but this a possibility that cannot be ruled out.

All results were adjusted for age and residential area according to the principle of control selection. In the analysis, we investigated the potential confounding from smoking and socio-economic group. Adjustment for these factors only marginally changed the estimated relative risks.

In the analysis, subjects who had been exposed to skin care products were compared with subjects with low use (score 0 to 20). In theory, the preferred comparison group should be totally unexposed (score 0). Among women, less than 0.6% was totally unexposed to cosmetics, which hampered the use of totally unexposed as a reference category. Among men, on the contrary, 15.9% were nonusers. We performed analyses comparing men exposed to cosmetics of various degrees with totally unexposed men. Still, no increased risks associated with cosmetic use were observed. Further, we did perform separate analyses regarding use of skin care products on different parts of the body (that is, face/neck, hands, legs, feet, chest/back) and risk of RA, since an association restricted to a specific part of the body may be hidden by the use of a global exposure measure. For none of the five specific parts of the body was an association seen.

To increase the interpretability of negative studies, as the current one, it is of interest to know the power of the study, or what magnitude of risk difference between the exposed and unexposed the study reasonably would detect. The size of the current study was sufficient (power > 0.80) to detect a risk increase in the order of 20 to 30% (for women and men taken together).

In 1998 we reported of induction of arthritis in the DA rat by common cosmetic products available at stores and pharmacies [16]. In the rat experiments we had chosen common commercial products with a general high share of mineral oil. The exact composition of the products, however, was not publicly available, neither in 1998 or today. The cosmetics, containing mineral oil, used in the experimental induction of arthritis, however, still contain mineral oil according to their actual declaration of ingredients. Besides, the most evident animal arthritis was induced by intradermal injection. The experimental arthritis induced by percutaneous administration on abrased skin was mild and of short duration; the corresponding human transient arthritis would very probably pass without any registration as early arthritis or RA. We therefore cannot exclude that a few individuals exposed to both skin trauma and certain mineral-oil-containing cosmetics may develop arthritis, but this is unlikely to be a common event.

We recently reported an association between occupational exposure to mineral oil and RF-positive RA and anti-CCP-positive RA, respectively, among men [18]. The male occupational exposure to mineral oil probably comprises both larger quantities and higher concentration of mineral oil than is the case during use of cosmetics, and, in addition, occupational exposure to mineral oil may often occur with simultaneous damage of the skin.

In summary, even though occupational exposure to mineral oil seems to be associated with an increased risk of RA, the use of skin care products - with contents of those during the late 1990s and before - does not seem to be a risk factor for RA in the population in general. Even though we cannot rule out that cosmetics can contribute to arthritis in some individuals carrying certain genotypes, our finding is thus of importance from a public health perspective, and there is no basis for a recommendation to avoid skin care products containing the usual amounts of mineral oil.

## Conclusion

This study does not support the hypothesis that ordinary usage of body lotions, skin creams or ointments, often containing mineral oil, increases the risk of RA in the population in general. We cannot exclude, however, the possibility that these cosmetics can contribute to arthritis in individuals carrying certain genotypes or simultaneously being exposed to other agents that contribute to the development of arthritis.

## Abbreviations

CCP: cyclic citrullinated peptide; EIRA: Epidemiological Investigation of Rheumatoid Arthritis; ELISA: enzyme-linked immunosorbent assay; RA: rheumatoid arthritis; RF: rheumatoid factor.

## Competing interests

The authors declare that they have no competing interests.

## Authors' contributions

BMS contributed to the hypothesis, the design of the study, and the interpretation and writing of the manuscript. HK performed a major part of the biostatistics work and contributed to the interpretation of results and the writing of the manuscript. LK and LA were responsible for the overall design of the EIRA study, for the analysis of data and the final writing of the manuscript. All authors read and approved the final text.
